# Response of Differently Structured Dental Polymer-Based Composites to Increasingly Aggressive Aging Conditions

**DOI:** 10.3390/nano15010074

**Published:** 2025-01-06

**Authors:** Nicoleta Ilie

**Affiliations:** Department of Conservative Dentistry, University Hospital, Ludwig-Maximilians-University, Goethestr. 70, D-80336 Munich, Germany; nicoleta.ilie@med.uni-muenchen.de

**Keywords:** nano-hybrid composites, fractography, three-point-bending test, strength, CAD/CAM, interpenetrating network

## Abstract

Objective: It is hypothesized that the way nano- and micro-hybrid polymer-based composites are structured and cured impacts the way they respond to aging. Material and methods: A polymer–ceramic interpenetrating network composite (Vita Enamic/VE), an industrially polymerized (Brillinat CriosST/BC), and an in situ light-cured composite with discrete inorganic fillers (Admira Fusion5/AF5) were selected. Specimens (308) were either cut from CAD/CAM blocks (VE/BC) or condensed and cured in white polyoxymethylene molds (AF5) and subjected to four different aging conditions (*n* = 22): (a) 24 h storage in distilled water at 37 °C; (b) 24 h storage in distilled water at 37 °C followed by thermal cycling for 10,000 cycles 5/55 °C (TC); (c) TC followed by storage in a 75% ethanol–water solution; and (d) TC followed by a 3-week demineralization/remineralization cycling. CAD/CAM samples were also measured dry before the aging process. Three-point bending test, quantitative and qualitative fractography, instrumented indentation test (IIT), SEM, and reliability analyses were used. Uni- and multifactorial ANOVA, Tukey’s post hoc test, and Weibull analysis were performed for statistical analysis. Results: A significant (*p* < 0.001) and very strong effect of the parameter material was observed (η_P_^2^ > 0.9). VE exhibited two to three times higher elastic moduli and hardness parameters compared to BC and AF5, which were comparable. Strength was highest in BC but was accompanied by high beam deformation. The effect of aging was comparatively smaller and was more evident in the IIT parameters than in the flexural strength or modulus. Reliability was high (m > 15) in VE and BC, regardless of aging protocol, while it was significantly reduced in AF5 following aging protocols b-d. Conclusions: TC was the method of artificial aging with a significant impact on the measured parameters, while demineralization/remineralization cycling had little or no impact. Clinical relevance: The degradation of composites occurred irrespective of the structuring and curing method and manifested in a low deterioration in the measured properties.

## 1. Introduction

Nano- and micro-hybrid polymer-based composites (composites) are used in dental practice primarily as light-curing paste materials to enable minimally invasive placing, individual aesthetic adaptation, and selective mechanical properties able to withstand in situ conditions. They have to confront the inherent presence of defects such as voids, fill-ers poorly integrated into the organic matrix, microstructural and polymerization hetero-geneities, filler agglomerates or inclusions that are either induced during their industrial production or are a result of layering and curing under clinical use. Regardless of their nature, these flaws can reach a critical size to propagate as a crack and finally to cause fracture [1]. 

In a chronologically more recent development, an improvement in the curing process was achieved through the industrial curing of the composites under controlled light, energy, heat, and pressure supply using computer-aided design–computer-aided manufacturing (CAD/CAM) blocks that would be later used clinically to mill a restoration [2]. The result was indeed an improvement in mechanical properties and more homogeneous polymerization, whereby the material-specific degree of variation in elasto-plastic mechanical behavior within a composite block is noticeable, while small, and estimated at less than 10% [3,4]. In addition to industrial curing, the restructuring of the build-up of the inorganic phase was designed, since the number of inorganic reinforcing particles that can be incorporated into the polymer matrix with acceptable porosity integration and filler agglomeration, and thus implicitly mechanical properties, is limited [5]. The inorganic phase of such materials no longer consists of dispersed micrometer-sized filler particles but a three-dimensional compact ceramic framework, which is later infiltrated with the polymer matrix and under laboratory conditions cured to form a CAD/CAM composite block. These polymer-infiltrated ceramic network (PICN) materials do not come close to the mechanical properties of dental ceramics (e.g., in terms of hardness or elastic modulus [6]) but exhibit high fracture toughness and rising R-curve behavior [7], i.e., increasing crack resistance with increasing crack length, which is associated with greater tolerance to contact and grinding-related damage [8].

The selection of appropriate restorative materials must also take into account aspects related to their longevity, durability, and degradation mechanisms under in situ conditions. Aging alters the mechanical properties, which in turn are responsible for the clinical performance of a restoration [9,10]. Regardless of the amount and organization of the filler system, the polymer matrix of composites consists of acrylates (e.g., bisphenol A glycol dimethacrylate (Bis-GMA); ethoxylated bisphenol A dimethacrylate (Bis-EMA); urethane dimethacrylate (UDMA) and its derivatives; and triethylene glycol dimethacrylate (TEGDMA)), which are all prone to degradation [11] under the harsh clinical conditions, and in particular to hydrolytic degradation due to ester, ether, urethane, and amide groups [12]. In addition, hydrolysis is catalyzed by salivary enzymes [12,13,14] as well as acids and bases, which can accelerate the reaction rate by several orders of magnitude [15]. Hydrolytic degradation is not limited to the polymer matrix, as it also affects the amphiphilic silane coupling agents used to connect fillers within the organic matrix [16].

The aim of the present study was therefore to define the impact of aging in relation to the way composites are structured and cured while exposing materials to various aging protocols, such as thermal cycling, ethanol immersion, and demineralization/remineralization cycling. Furthermore, the response to aging should be quantified at macro and micro levels as well as in static and dynamic approaches.

The null hypotheses tested state that aging, which consists of either storage in distilled water at 37 °C for 24 h or thermal cycling for 10,000 cycles between 5 and 55 °C, thermal cycling followed by storage in a 75% ethanol–water solution for 72 h, or thermal cycling followed by a 3-week demineralization/remineralization cycling, affects different structured and cured composites similarly in terms of (a) mechanical properties and fracture behavior measured at a macroscopic scale (flexural strength and modulus, fracture pattern, location of critical flaw, mirror constant, reliability) and (b) elastoplastic behavior, evaluated at a microscopic scale.

## 2. Materials and Methods

### 2.1. Materials

Three dental polymer-based composites based on different curing procedures, chemical compositions, and microstructures were employed (Table 1): (1) a nano-hybrid light-curing composite, Ormocer-based (organically modified ceramic) with silica and barium-alumino-boro-silicate glass fillers (AF5) that was cured for 10 s with a violet–blue Light Emitted Diode Light-Curing Unit (LED-LCU: Bluephase^®^ Style, Ivoclar Vivadent, Schaan, Lichtenstein; 1400 mW/cm^2^; violet peak at 412 nm and blue peak at 455 nm); (2) a super translucent CAD/CAM polymer-based composite (BC) with a comparable filler system as AF5 but lower filler amount; and (3) a polymer-infiltrated ceramic network (PICN) CAD/CAM composite.

### 2.2. Methods

Composite specimens were subjected to four different aging conditions: (a) 24 h storage in distilled water at 37 °C; (b) 24 h storage in distilled water at 37 °C followed by thermal cycling for 10,000 cycles 5/55 °C (abbreviated as TC); (c) TC followed by storage in a 75% ethanol–water solution for 72 h (TC + EtOH); and (d) TC followed by a 3-week of demineralization/remineralization cycling (TC + de/rem). In addition, CAD/CAM samples were also measured dry before the aging process. A three-point bending test, quantitative and qualitative fractography, instrumented indentation tests (IITs), Fe-SEM, and reliability analyses were employed.

#### 2.2.1. Specimen Preparation and Aging Conditions

Briefly, 308 parallelepiped slabs (2 mm × 2 mm × 18 mm, *n* = 22) were prepared. The composite AF5 was placed in a white polyoxymethylene (POM) mold and cured while following the dental standards ISO 4049:2019 [17] and NIST No. 4877 [18]. The samples were polymerized on the top and bottom for 10 s. Therefore, three light exposures were applied, taking care not to overlap the irradiated sections by more than 1 mm of the light guide diameter. Directly after curing, the specimens were detached from the mold and stored in distilled water at 37 °C for 24 h. The four largest surfaces of the samples of the 88 AF5 specimens were individually wet ground (EXAKT 400CS Micro Grinding System EXAKT Technologies Inc., Oklahoma City, OK, USA) with consecutively finer abrasive paper (silicon carbide: P1200, P2500, P4000). In addition, for each of the CAD/CAM composites, 110 specimens of similar geometry were cut out from blocks and wet ground as described above to obtain 2 mm × 2 mm × 18 mm with an accuracy of 0.1 mm. Of these, 22 specimens per CAD/CAM composite were kept dry, while the rest were stored in distilled water at 37° C for 24 h. For each material, 22 specimens were tested after 24 h storage; the remainder were additionally thermally aged (TC). For this purpose, 10,000 cycles between 5 and 55 °C were carried out, using distilled water as the immersion medium (Willytec, Dental Research Division, Munich, Germany). Again, 22 specimens per material were tested, while the remainder were exposed to either a 75% alcohol–water mixture for 72 days (TC + EtOH) or to a similar protocol to that used for the demineralization/remineralization of tooth structure (TC + de/rem). The latter protocol consists of 15 demineralization/remineralization cycles, each requiring 8 h of storage in a demineralization solution (1.5 mM CaCl_2_, 0.9 mM KH_2_PO_4_, 5 mM NaN_3_, and 50 mM CH_3_COOH per 1 L distilled water; pH = 4.8 adjusted with NaOH), followed by storage in a remineralization solution for 16 h (1.5 mM CaCl_2_, 0.9 mM NaH_2_PO_4_, 0.13 M KCl, 5 mM NaN_3_, and 20 mM C_8_H_18_N_2_O_4_S (HEPES), pH = 7.0 adjusted with NaOH). The demineralization/remineralization cycling took place on weekdays, while on weekends the specimens were permanently kept in the remineralization solution.

#### 2.2.2. Three-Point Bending Test (3-PBT)

A 3-point bending test was employed according to NIST No. 4877 [18] to determine the strength (FS), modulus of elasticity (E), and beam deflection (ɛ) while using a span of 12 mm. The samples described above were placed in a universal testing machine and loaded at a crosshead speed of 0.5 mm/min until fracture (Z 2.5 Zwick/Roell, Ulm, Germany).

#### 2.2.3. Fractography Analysis

A stereomicroscope (Stemi 508, Carl Zeiss AG, Oberkochen, Germany) was used to analyze all fractured samples. A total of 616 surfaces were examined from 308 samples, which were divided into two parts. The part with the clearest fractographic traces was photographed and evaluated with a microscope extension camera (Axiocam 305 color, Carl Zeiss AG, Oberkochen, Germany). The location of the fracture origin (volume or surface defects) and the fracture pattern represent qualitative fractography, while the fracture mirror and the fracture mirror radius enabled quantitative fractography to be performed. Care was taken to identify the mirror boundaries that were associated with the first noticeable roughness that followed the smooth mirror area. After identifying these regions, the radius of the mirror (*R*) running in the direction of constant stress, i.e., parallel to the tensile side of the sample, was measured (ImageJ Version 1.53 k, U.S. National Institutes of Health, Bethesda, MD, USA). In the event that the origin of the fracture was not clearly identifiable, the diameter of the mirror was measured and then halved.

The parameters strength (*σ*) and mirror radius (*R*) were used in the calculation of the mirror constant (*A*), as described by the Orr equation [19]:

(1) $$\sigma \sqrt{R}=A$$

#### 2.2.4. Field-Emission Scanning Electron Microscopy

Additionally, specimens (*n* = 3) were prepared for each material as described above to determine the structural appearance of the filler system while using an electron backscatter diffraction approach (field-emission scanning electron microscopy, Zeiss Supra 55VP, Carl Zeiss GmbH, Göttingen, Germany).

#### 2.2.5. Instrumented Indentation Test (IIT): Quasi-Static Indentation Test

A total of 70 samples, with 5 randomly selected samples per material and aging conditions resulting from the 3-PBT, were subjected immediately following testing to a quasi-static instrumented indentation test (IIT) as described in ISO 14577 [20] (Fischerscope^®^ HM 2000, Helmut Fischer GmbH, Sindelfingen, Germany). Six measurements were randomly performed on each sample. Indentation (Vickers diamond tip) occurred force-controlled. For this purpose, the test load increased from 0.4 mN to 2000 mN at a constant speed within 20 s, was held at the maximum force for 5 s, and then decreased within 20 s. The variation in the indentation force (F) and indentation depth (h) during the above-described indentation cycle was used to calculate a battery of parameters able to assess the elastic and plastic material behavior. These included the indentation modulus E_IT,_ which involved the slope of the tangent of the F-h curve at the maximum force. In addition, the hardness with its plastic and elastic components was calculated by accounting for the impression created during the indentation while differentiating between the projected indenter contact area (A_c_) and the surface area of the indentation under the applied test load (A_s_). A_c_ was determined from the F-h curve as specified in ISO 14577 [20]. The resistance to plastic deformation was defined by the indentation hardness (H_IT_ = F_max_/A_c_) and converted to its more familiar correspondent, the Vickers hardness (HV = 0.0945 × H_IT_). The universal hardness (equivalent to Martens hardness = F/A_s_(h)) was calculated by dividing the test load by A_s_ and involves both plastic and elastic deformation. The changes in the indentation depth during the time in which the maximal indentation force was maintained for 5 s defined the creep.

### 2.3. Statistical Analyses

The variables were normally distributed (Shapiro–Wilk method), and the homogeneity of variance was confirmed. One- and multiple-way analysis of variance (ANOVA) and Tukey’s honest significant difference (HSD) tests post hoc test using an alpha risk set at 5% were therefore used for analysis (SPSS Inc. Version 29.0, Chicago, IL, USA).

FS data were also used in a Weibull analysis [21]:

(2) $$P_{f}(\sigma_{c})=1-\exp\left[-{\left(\frac{\sigma_{c}}{\sigma_{0}}\right)}^{m}\right]$$

where $\sigma_{c}$ is the measured strength; *m* is the Weibull modulus; and $\sigma_{0}$ is the characteristic strength, defined as the uniform stress at which the probability of failure is 0.63. After rearranging and considering the double logarithm of Equation (2), plotting ln ln(1/(1 − *P*)) versus ln$\sigma_{c}$ yields a straight line, with the upward gradient m defined as the Weibull modulus or reliability [21].

## 3. Results

A multifactorial analysis showed a significant (*p* < 0.001) and very strong influence of the material on the measured properties, with a slightly larger influence on the micromechanical parameters compared to the macromechanical parameters (higher partial eta-squared values, η_P_^2^, Table 2a,b). The influence of aging was comparatively less, but the difference in the impact increased sharply from the macro- to the micromechanical parameters. Also, the binary effect of the main parameters (composite × aging) was significant.

**Figure 1:**
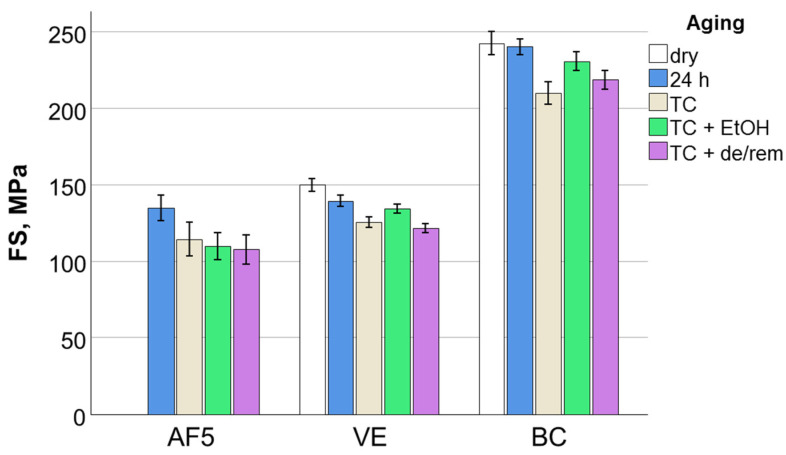
Flexural strength (FS) as a function of material and aging conditions.

The results of the 3-PBT are presented in Table 3 and Figure 1. The flexural strength and the mirror constant A decreased significantly across all storage conditions in the material sequence BC > VE > AF5. In contrast, the flexural modulus was two to three times higher for VE, followed by BC and then AF5, while the beam deflection followed the order BC > AF5 > VE.

**Figure 2:**
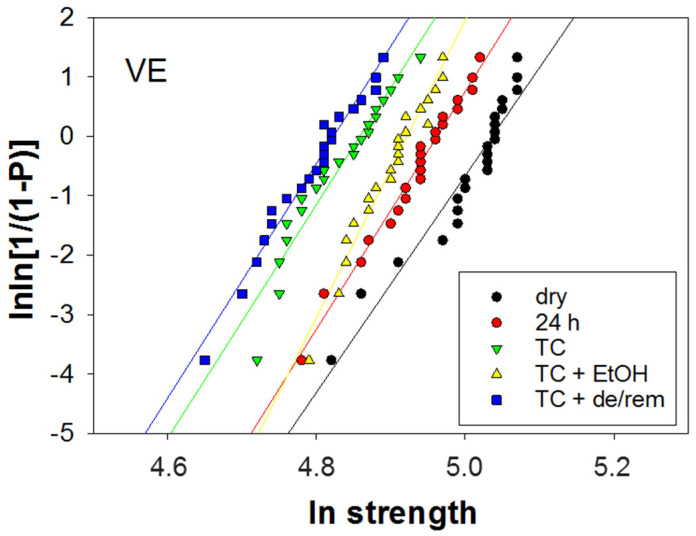
The Weibull plot representing the empirical cumulative distribution function of strength data exemplified for VE as a function of aging condition. The goodness of fit was assessed by linear regression.

**Figure 3:**
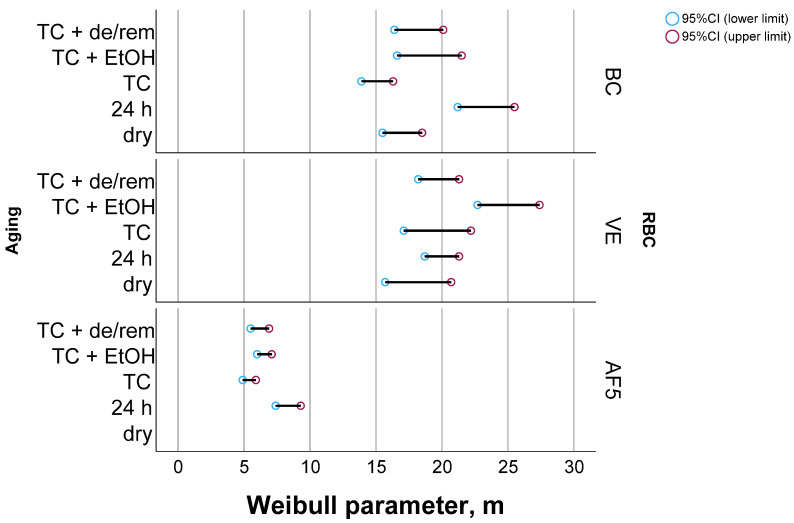
The Weibull parameter with lower and upper limits of the 95% confidence interval, as a function of material and aging condition.

Figure 2 shows a Weibull diagram exemplified for the material VE as a function of the aging condition, while Figure 3 summarizes the Weibull parameter m with the 95% confidence interval for all materials and aging conditions. The material reliability, expressed by the Weibull parameter, m, was comparable and very high in BC and VE, while it was lower in AF5. A significant difference in the Weibull parameter was observed when the confidence intervals did not overlap. Accordingly, thermal aging significantly reduced the reliability in AF5 compared to the 24 h storage, while the additional aging protocols preserved the TC values. In BC, the 24 h storage increased the reliability compared to the dry condition, while the other aging conditions returned the reliability to the original value. The smallest influence of aging on reliability was observed for VE, whereas only ethanol storage slightly improved the Weibull parameter at the border of the significance level.

**Figure 4:**
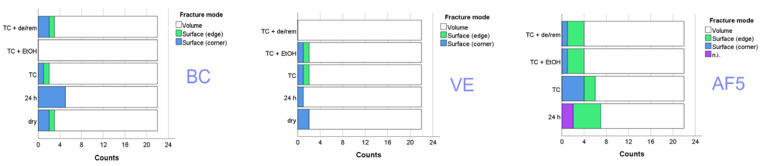
Fracture mode distribution among analyzed materials and aging treatment (n. i. = not identifiable failure mode).

Qualitative fractography revealed that failures initiated by volume defects were the prevalent failure mode (86.7%) with surface defects accounting for 5.8% (edge) and 6.8% (corner), while 0.7% of the failures were not clearly identifiable (n. i.). Figure 4 shows the distribution of the fracture modes within each material and aging condition.

VE exhibited two to three times higher indentation modulus and hardness parameters compared to BC and AF5, while the material sequence in descending order with respect to HM, HV, and E_IT_ was VE > AF5 > BC and inverse for creep (Table 3).

**Figure 5:**
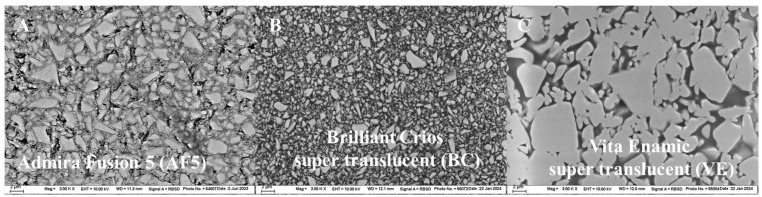
SEM evaluation of the analyzed filler systems at 3k magnification (**A**) AF5; (**B**) BC; (**C**) VE.

The structural appearance of the filler system is shown in Figure 5 at 3k magnification. Compact, geometrically irregular, split fillers are identified in AF5 and BC, with larger fillers observed in the former case. In VE, the ceramic framework can be observed, which is filled with the darker-appearing polymer.

## 4. Discussion

The long-term clinical behavior of dental restoration is a difficult matter to predict. The criticism of using the initial material parameters for such predictions and ignoring their aging behavior is therefore entirely justified. Various tests and simulated environmental conditions are required, and initial material parameters should only be used to screen out clinically unsuitable materials. In this context, it is important to note that the development of modern composite materials has already reached a very high standard and level of maturity, reflected in good clinical performance. In this line, the cumulative survival rates were estimated at 91.7% after 6 years of serving, 81.6% after 12 years, and 71.4% after 29 years [22]. The battle for longevity compared to amalgam fillings also seems to have been won, as composite fillings have clinically proven to be at least comparable, if not significantly superior [23].

The present study assessed the effect of aging on material properties at various scales. The decision to use a macroscopic mechanical test to assess composite degradation was motivated by positive correlation analyses between the flexural strength of aged composites and the clinical performance of composite restorations [24]. Particular attention has been paid to surface treatment as it determines the interaction of a composite with its environment, with rough surfaces potentially forming spots that can initiate cracks, accelerate wear, and increase the coefficient of friction compared to smooth surfaces [25]. All four large sides of each parallelepiped sample were therefore processed in a standardized manner, making sample preparation for this type of test time-consuming. Additionally, the same surface finishing process was used across all materials tested to support the intent of mimicking clinical applications where direct and indirect composite restorations require high polishing. The instrumented indentation test performed at the microscopic scale has not yet been correlated with clinical performance but has been found to be more sensitive than flexural strength in the early detection of aging-related changes in dental composites [4], a fact confirmed in the present study. The test also offers a variety of parameters that allow for the characterization of both elastic and plastic deformation.

An Ormocer-based composite (organically modified ceramics [26]), which is based on a pre-crosslinked inorganic Si-O-Si network prepared by hydrolysis and polycondensation reactions [27], was selected as the light-curing composite. Multifunctional urethane and thioether (meth)acrylate alkoxysilanes then crosslinked the inorganic Si-O-Si network in the polymerization reaction [28]. With an inorganic filler content of 84% by weight, the material is one of the most highly filled light-curing composites, which enables a fair comparison with the industrially cured materials. The irregular inorganic fillers used are larger compared to the industrially cured material BC, both materials with discrete inorganic fillers. However, the advantage of a higher filler amount and size was not reflected in an improved elastic modulus at the macroscopic level as expected but was only evidenced by a slight advantage in the indentation modulus at the microscopic level. The mechanical reinforcement mechanism of composites must be considered as a multifactorial and complex event, which, in addition to the more obvious factors such as the amount and size of inorganic fillers, also includes the silanization of the fillers [29] and the organization (e.g., percolation [30] and crosslinking [31]) of the polymer matrix.

In addition to the large difference in the modulus of elasticity between an inorganic glass filler and the methacrylate polymer, from a microscopic perspective, macromolecules adhere to the inorganic particles via silanes, forming a bonded, immobile polymer layer around particles with a higher modulus of elasticity than the base organic matrix [30]. This fact also contributes to improvement in the mechanical properties. Both the increase in the number of particles and the decrease in the particle size led to an increase in the amount of boundary layer and their ratio and influence on the total volume of the organic matrix. Both analyzed composites with discrete inorganic fillers were highly filled, involving a very small distance between the particles (Figure 5A,B) such that the boundary layers adjacent to the fillers described previously touched and impacted each other, resulting in the percolation of the polymer phase attached to the fillers. A further consequence of this circumstance is that the deformability of the polymer matrix is locally greatly reduced [30], since there is little or no polymer material in between the fillers to trigger such deformation processes, which, among other things, explains the brittle character of composite materials in spite of the high monomer content. Therefore, the smaller filler size in BC can compensate for the smaller amount of filler compared to AF5 to some extent. In addition, due to the nature and enlargement of macromolecules, Ormocers are characterized by low crosslinking and a low degree of cure, which are further effects responsible for the lower elastic modulus [32].

The presence of a filler in a polymer matrix as a local inhomogeneity, together with the above-described changes in the properties of the organic matrix bound to a filler [30], hinders a crack or requires more energy for its formation, growth, and propagation [33], which increases the fracture toughness. Qualitative and quantitative fractography can be used to evaluate this parameter in brittle materials, including highly filled composites [1,34]. After a fracture is initiated from a critical flaw liable to propagating as a crack [1], it leaves behind a characteristic pattern defined by an initial smooth radial region, the fracture mirror [35], that is delimitated by a slightly rougher region termed mist. The latter arises from secondary cracks that are no longer able to propagate as energy decreases [36] when the crack tip deviates from the main plain. The mist region then transits into a region of larger radial ridges, the hackle lines, which leads to macroscopic crack branching [37]. The radius of the fracture mirror measured along the tensile surface [38] can then be used to calculate a material parameter, the mirror constant (A), as described in Material and Methods, which has been shown to correlate with the critical fracture toughness K_Ic_ [38]. When comparing the materials with particulate fillers, the mirror constant A was higher in BC than in AF5, which can be explained by two lines of reasoning: firstly as the confirmation of the Griffith energy criterion, which directly links fracture toughness to E [39] and secondly by the smaller filler sizes in BC, which provide a larger filler–matrix interface. Since the latter is the weakest link in a composite, the propagating crack tip can be blunted while retarding crack growth as the filler debond from the organic matrix, allowing for an increase in fracture toughness. Energy dissipation through debonding can also delay crack growth because the driving force at the crack tip decreases [33]. As for the polymer–ceramic interpenetrating network composite VE, the consistently higher E was not directly reflected in the value of the mirror constant, which was intermediate between the two discrete filler composites. While the feldspar ceramic skeleton provided rigidity and high resistance to deformation, the interface between ceramic and polymer was lower compared to the composite with particulate fillers surrounded by the polymer, providing less potential for energy dissipation and crack growth retardation. The lower fracture toughness of VE compared to BC has also been documented in the literature, confirming these results.

The scatter of the measured strength data was well modeled by the Weibull analysis in all materials and aging methods, which was confirmed by the very high R^2^ values (>0.94). The reliability (Weibull parameter m) of VE and BC was statistically similar and very high, while the large majority of specimens failed due to volume defects. The significantly lower reliability observed with AF5, and in particular the fact that it was the only material whose reliability decreased with aging, must be attributed to the light-curing versus the industrial curing of the other materials. Volume defects as a reason for the initiation of fracture were also predominant in AF5, although a slightly higher proportion of surface defects were noticeable compared to the other two materials. Since all materials underwent identical surface preparation, this fact can be clearly associated with the larger filler sizes in AF5 and thus the higher probability of developing a critical defect size that can trigger fracture due to the higher intrinsic roughness. Another benefit of the calculated Weibull parameters is that they allow for the conversion of the strength values measured under NIST No. 4877 conditions to values corresponding to ISO 4049 [40], as the smaller size of the CAD/CAM blocks did not allow sample preparation with a length of 25 mm.

The degradation of dental composites usually occurs through hydrolytic processes, which can be accelerated by environmental conditions and factors such as temperature, pH, and especially enzymes, as described in the Introduction. Immersion media diffuse through the material and lead to plasticization, the softening and swelling of the polymer matrix, the leaching of filler components, and the degradation of the adhesion promoter. Interestingly, while the analyzed materials underwent deterioration during aging, particularly after thermal aging, the decrease in the measured mechanical parameters was surprisingly low. This must certainly be related to adequate polymerization both industrially for the CAD/CAM composites and in the laboratory for AF5 by providing the required radiant exposure under controlled and standardized conditions. Apart from the pH value of the degradation medium, the amount and rate of water uptake [31] are relevant factors for the degradation of a polymer matrix [13] and the silane coupling agent [41]. In addition to adequate polymerization, both CAD/CAM composites contained UDMA, a monomer with lower water sorption compared to Bis-GMA, which is currently still the most commonly used monomer in dental composites. Ormocers are also characterized by low water sorption. While several studies involve the use of a strongly alkaline pH to induce the degradation of composite in vitro, the method does not correspond to clinical conditions. It is conceivable that the pH value is more acidic, which was the deciding factor in following a protocol that is used in the laboratory to create artificial caries. The simulated conditions did not have a serious impact on the measured parameters in any of the tested materials.

## 5. Conclusions

The mechanical behavior of a composite material and its altering during various aging protocols is a complex process that depends on the amount, size, distribution, and interrelation of the individual constituent components. The durability of the physical properties decreased upon exposure to water for all materials regardless of the type of curing or microstructure but was comparatively low and manifested more clearly at the microscale. Only the light-curing composite lost reliability as it aged. Thermal cycling was the method of artificial aging with a significant impact on the measured parameters, while demineralization/remineralization cycling had little or no impact. The ranking of materials for a given parameter was maintained regardless of the aging condition.

## Figures and Tables

**Table 1:** Composition and characteristics of the analyzed polymer-based composites.

Material/Manufacturer	LOT	Composition		wt%	vol%
		Matrix	Filler		
Admira Fusion 5 (**AF5**) Voco, Cuxhafen, Germany	V97552	Ormocer	BaO-Al_2_O_3_-B_2_O_3_-SiO_2_; SiO_2_	84	-
Brilliant Crios super translucent (**BC**) Coltene, Altstätten, Switzerland	K82874	BisGMA UDMA TEGDMA	BaO-Al_2_O_3_-SiO_2_; SiO_2_	70.7	51.5
Vita Enamic super translucent (**VE**) VITA	74770	UDMA TEGDMA	feldspar ceramic network	86	75

Abbreviations: Ormocer = organically modified ceramic; BisGMA = bisphenol A glycol dimethacrylate; UDMA = urethane dimethacrylate; TEGDMA = triethylene glycol dimethacrylate; SiO_2_ = silicon oxide (silica); BaO-Al_2_O_3_-SiO_2_ = barium-alumino-silicate glass; BaO-Al_2_O_3_-B_2_O_3_-SiO_2_ = barium-alumino-boro-silicate glass; wt% = percent by weight; vol% = percent by volume; “-” = not stated.

**Table 2:** (**a**) Effect strength of the main parameters—composite and aging—and their interaction product on the measured parameters. Partial eta-squared values (η_P_^2^) are indicated whenever the effect is significant *p* < 0.001); (**b**) effect strength of aging on the measured parameters within each material. Partial eta-squared values (η_P_^2^) are indicated whenever the effect is significant *p* < 0.001).

(**a**)								
**Parameter**	**E** **GPa**	**FS** **MPa**	**ɛ** **%**	**A** **MPa m^1/2^**	**HM N/mm^2^**	**HV** **N/mm^2^**	**E_IT_/(1 − vs^2^)** **GPa**	**Cr** **%**
Composite	0.95	0.91	0.94	0.55	0.99	0.99	0.99	0.95
Aging	0.17	0.29	0.16	0.06	0.50	0.39	0.57	0.34
Composite × Aging	0.14	0.08	0.20	0.11	0.36	0.24	0.46	0.18
(**b**)								
**Parameter**	**E** **GPa**	**FS** **MPa**	**ɛ** **%**	**A** **MPa m^1/2^**	**HM N/mm^2^**	**HV N/mm^2^**	**E_IT_/(1 − vs^2^)** **GPa**	**Cr** **%**
AF5	0.56	0.15	0.26	0.23	0.78	0.86	0.65	0.41
BC	0.60	0.42	0.34	-	0.79	0.81	0.69	0.51
VE	0.19	0.63	0.17	-	0.55	0.41	0.70	0.38

Abbreviations: E = flexural modulus; FS = flexural strength; ε = beam deflection; A = mirror constant; HM = Martens hardness; HV = Vickers hardness; E_IT_ = indentation modulus; Cr = creep.

**Table 3:** Variation in the measured parameters with composite and immersion condition (TC = thermal cycling; EtOH = 75% alcohol–water mixture, de/rem = de- and remineralization protocol; SD = standard deviation).

Composite	Aging		3-Point Bending Test				Instrumented Indentation Test			
			E GPa	FS MPa	ε %	A MPa√m	HM N/mm^2^	HV N/mm^2^	E_IT_ GPa	Cr %
BC	dry	Mean	8.9	242.1	3.6	2.5	541.5	82.6	11.6	4.4
		SD	0.5	17.1	0.5	0.4	25.9	4.0	0.6	0.1
	24 h	Mean	8.1	239.9	4.2	2.8	519.1	78.1	11.3	4.5
		SD	0.4	12.1	0.4	0.3	7.2	1.4	0.2	0.1
	TC	Mean	7.6	209.8	3.8	2.5	491.1	74.2	10.6	4.5
		SD	0.5	16.7	0.6	0.3	6.9	0.7	0.3	0.1
	TC + EtOH	Mean	7.5	230.4	4.5	2.6	468.3	69.6	10.2	4.8
		SD	0.3	14.1	0.5	0.3	9.0	1.7	0.2	0.2
	TC + de/rem	Mean	7.3	218.2	4.2	2.8	512.5	77.4	11.0	4.6
		SD	0.7	14.3	0.5	0.3	6.9	1.1	0.2	0.1
VE	dry	Mean	17.8	149.7	0.8	2.2	1540.0	268.3	30.3	2.7
		SD	1.5	9.2	0.1	0.3	60.4	12.9	1.1	0.1
	24 h	Mean	18.1	139.1	0.8	2.1	1442.2	253.6	27.8	2.9
		SD	1.3	8.3	0.1	0.3	50.0	11.1	1.0	0.2
	TC	Mean	16.5	125.2	0.7	2.0	1349.0	236.3	25.9	3.1
		SD	2.5	7.6	0.1	0.3	45.8	11.4	0.7	0.2
	TC + EtOH	Mean	16.6	134.3	0.8	2.2	1414.2	246.3	27.4	3.1
		SD	1.3	6.5	0.1	0.3	75.8	17.1	1.1	0.1
	TC + de/rem	Mean	17.1	121.2	0.7	2.0	1428.9	248.4	27.9	2.9
		SD	1.4	7.2	0.1	0.3	52.4	12.2	0.8	0.2
AF5	24 h	Mean	6.6	134.5	2.2	1.8	604.5	98.1	11.9	4.3
		SD	0.8	18.6	0.5	0.2	29.3	2.2	1.0	0.1
	TC	Mean	7.4	114.2	1.6	1.9	630.3	100.5	12.7	4.2
		SD	0.3	24.6	0.4	0.3	17.6	2.3	0.5	0.0
	TC + EtOH	Mean	6.3	109.7	1.8	2.0	546.3	86.2	11.1	4.3
		SD	0.4	19.7	0.4	0.3	24.8	4.0	0.6	0.2
	TC + de/rem	Mean	7.9	107.5	1.4	1.6	658.4	104.3	13.5	4.1
		SD	0.4	21.2	0.3	0.3	16.6	2.8	0.4	0.2

Abbreviations: E = flexural modulus; FS = flexural strength; ɛ = beam deflection; A = mirror constant; HM = Martens hardness; HV = Vickers hardness; E_IT_ = indentation modulus; Cr = creep.

## Data Availability

Data is available on request.
